# Detection of lymph node metastasis of oesophageal cancer by RT-nested PCR for SCC antigen gene mRNA

**DOI:** 10.1054/bjoc.1999.0938

**Published:** 2000-01-18

**Authors:** M Kano, Y Shimada, J Kaganoi, T Sakurai, Z Li, F Sato, G Watanabe, M Imamura

**Affiliations:** Surgery and Surgical Basic Science and Laboratory of Anatomic Pathology, Graduate School of Medicine, Kyoto University, 54 Shogoin Kawara-cho, Sakyo-ku, Kyoto, 606-8397, Japan

**Keywords:** oesophageal cancer, SCC antigen, RT-PCR

## Abstract

With recent development in molecular biology, reverse transcriptase polymerase chain reaction (RT-PCR) has been applied to detect occult lymph node metastasis, but there have been few reports concerning oesophageal cancer. The objective of this study is to investigate the usefulness of the squamous cell carcinoma (SCC) antigen gene as a marker with RT-nested PCR to detect occult lymph node metastases of oesophageal cancer. The SCC antigen has been widely used as a serum tumour marker and was reported as a target gene to detect tumour cells in peripheral blood in cervical cancer. In this study, 620 lymph nodes from 14 oesophageal cancer patients were analysed. The results of RT-nested PCR were compared with that of pathological and immunohistochemical examinations. In the test of sensitivity, the RT-nested PCR detected 10^1^of SCC antigen producing cells in 10^7^peripheral blood mononucleocytes and was not found in 43 control lymph nodes. The pathological examination, immunohistochemical examination and the RT-nested PCR detected 36, 45 and 65 nodes respectively. The RT-nested PCR detected statistically more lymph nodes than the pathological or immunohistochemical examination. The sensitivity and specificity seem higher in squamous cell carcinoma cases. The SCC antigen gene is one of the more useful markers for RT-nested PCR to detect occult lymph node metastases of oesophageal cancer. © 2000 Cancer Research Campaign


*British Journal of Cancer* (2000) **82**(2), 429–435
© 2000 Cancer Research Campaign
Article no. bjoc.1999.0938

### Detection of lymph node metastasis of oesophageal cancer by RT-nested PCR for SCC antigen gene mRNA

**M Kano[1], Y Shimada[1], J Kaganoi[1], T Sakurai[2], Z Li[1], F Sato[1], G Watanabe[1] and M Imamura[1]**

[1]Surgery and Surgical Basic Science, Graduate School of Medicine, Kyoto University and [2]Laboratory of Anatomic Pathology, Kyoto University Hospital, 54 Shogoin Kawara-cho, Sakyo-ku, Kyoto, 606-8397, Japan

**Summary** With recent development in molecular biology, reverse transcriptase polymerase chain reaction (RT-PCR) has been applied to detect occult lymph node metastasis, but there have been few reports concerning oesophageal cancer. The objective of this study is to investigate the usefulness of the squamous cell carcinoma (SCC) antigen gene as a marker with RT-nested PCR to detect occult lymph node metastases of oesophageal cancer. The SCC antigen has been widely used as a serum tumour marker and was reported as a target gene to detect tumour cells in peripheral blood in cervical cancer. In this study, 620 lymph nodes from 14 oesophageal cancer patients were analysed. The results of RT-nested PCR were compared with that of pathological and immunohistochemical examinations. In the test of sensitivity, the RT-nested PCR detected $10^1$ of SCC antigen producing cells in $10^7$ peripheral blood mononucleocytes and was not found in 43 control lymph nodes. The pathological examination, immunohistochemical examination and the RT-nested PCR detected 36, 45 and 65 nodes respectively. The RT-nested PCR detected statistically more lymph nodes than the pathological or immunohistochemical examination. The sensitivity and specificity seem higher in squamous cell carcinoma cases. The SCC antigen gene is one of the more useful markers for RT-nested PCR to detect occult lymph node metastases of oesophageal cancer. © 2000 Cancer Research Campaign

**Keywords:** oesophageal cancer; SCC antigen; RT-PCR

In oesophageal cancer, as in many other malignancies, lymph node metastasis is an important prognostic factor (Skinner et al, 1982; Sugimachi et al, 1986; Siewert and Roder, 1992; Kato et al, 1993; Baba et al, 1994, 1997; Fahn et al, 1994; Roder et al, 1994; Nishimaki et al, 1994; Bhansali et al, 1997). Of patients who undergo curative surgery, 13.6–51% of patients relapse within 5 years (Abe et al, 1990; Fahn et al, 1994; Morita et al, 1994; Law et al, 1996; Bhansali et al, 1997). Therefore accurate evaluation of cancer spread is desirable to assess both the risk of recurrence and the prognosis.

Recent developments in molecular biology have produced new methods to detect smaller numbers of tumour cells. Many kinds of molecular markers to detect occult tumour cells are being sought (Pelkey et al, 1996; Raj et al, 1998). It is still controversial whether occult metastases found by recent techniques have an impact on patients' prognosis or not. As for oesophageal cancer, Izbicki et al (1997) and Luketich et al (1998) reported that microlymph node metastasis indicates poor prognosis. On the other hand, Glickman et al (1999) do not recommend extensive lymph node sectioning with keratin immunohistochemistry because occult lymph node metastasis was not an independent poor prognostic feature in their study.

The squamous cell carcinoma (SCC) antigen has been widely applied as a serum tumour marker of SCC of the uterine cervix, oesophagus, head and neck, and non-small-cell lung cancer and some other organs (Kato, 1992). Recently, Stenman et al (1997) succeeded in detecting tumour cells in the blood of patients with cervical SCC by RT-nested PCR for SCC antigen gene mRNA.

In this study, we compared the result of the RT-nested PCR for the SCC antigen mRNA with that of the pathological and the immunohistochemical examination for cytokeratins to investigate the usefulness of the SCC antigen gene mRNA expression as a marker to detect micrometastases of oesophageal cancer.

*Received*
*Revised*
*Accepted*

*Correspondence to:* M Kano

### MATERIALS AND METHODS

### Lymph node and tissue samples

A total of 620 lymph nodes were obtained from 14 patients with oesophageal carcinoma who underwent oesophagectomy at Kyoto University Hospital (Table 1). Twelve patients had SCC, one patient had adenocarcinoma originating from the Barrett's epithelium and one patient had basaloid squamous carcinoma. Three pN0 patients were included in this study. Normal and malignant oesophageal tissues were taken from surgical specimens immediately after resection and stored in liquid nitrogen until the extraction of RNA. Each lymph node was serially numbered and then cut into two pieces. One half of a lymph node was formalin-fixed and subjected to the routine pathological and immunohistochemical examination. The other half was snap-frozen in liquid nitrogen and kept in liquid nitrogen until the extraction of RNA. We used disposable surgical scalpels to cut lymph nodes to avoid cross-contamination. As controls, lymph nodes were obtained from breast cancer, gastric cancer, colon cancer and rectal cancer patients. Each control node proved to be negative for metastasis by pathological examination and each primary tumour was confirmed to be negative for the SCC antigen gene expression by RT-nested PCR.

**Table 1** Clinicopathological background of 14 patients

| No. | Age | Sex | Localization | Histological type | pT | pN | pM | pTNM stage | Serum SCC (ng ml$^{-1}$) |
|---|---|---|---|---|---|---|---|---|---|
| 1 | 60 | F | Ce | Mod. | pT2 | pN1 | pM0 | 2B | 2.7 |
| 2 | 58 | M | Ce | Well | pT4 | pN1 | pM0 | 3 | <0.5 |
| 3 | 62 | M | Ce | Poor. | pT2 | pN1 | pM0 | 2B | 2.3 |
| 4 | 53 | M | Iu | Mod. | pT3 | pN1 | pM1a | 4A | 1.9 |
| 5 | 62 | M | Im | Well | pT1 | pN0 | pM0 | 1 | 0.69 |
| 6 | 67 | M | Im | Mod. | pT1 | pN1 | pM0 | 2B | 0.87 |
| 7 | 48 | F | Im | Basaloid | pT3 | pN1 | pM0 | 3 | 0.73 |
| 8 | 61 | M | Ei | Mod. | pT3 | pN1 | pM0 | 3 | 3.9 |
| 9 | 64 | M | Ei | Well | pT3 | pN0 | pM0 | 2A | 0.77 |
| 10 | 50 | M | Ei | Poor. dif. adeno. ca. | pT3 | pN1 | pM0 | 3 | <0.5 |
| 11 | 50 | M | Ei | Mod. | pT2 | pN1 | pM0 | 2A | <0.5 |
| 12 | 56 | M | Ei | Well | pT2 | pN1 | pM0 | 2A | 15.4 |
| 13 | 66 | M | Ei | Mod. | pT2 | pN1 | pM0 | 2A | 0.84 |
| 14 | 71 | M | Ei | Well | pT2 | pN0 | pM0 | 2A | 3.5 |

Ce: cervical oesophagus, Iu: upper thoracic oesophagus, Im: middle thoracic oesophagus, Ei: lower thoracic oesophagus, Well: well differentiated squamous cell carcinoma, Mod.: moderately differentiated squamous cell carcinoma, Poor.: poorly differentiated squamous cell carcinoma, Poor. dif. adeno. ca.: poorly differentiated adenocarcinoma, Basaloid: basaloid (-squamous) carcinoma.

### Cell line and normal peripheral blood mononucleocytes

The oesophageal cancer cell line, KYSE 273 (Shimada et al, 1992) that was established from an oesophageal SCC in our laboratory, was used in this study. This cell line produces SCC antigen in its culture medium (Shimada, 1999). Normal peripheral blood mononucleocytes (PBMN) were harvested from the heparinized blood of healthy volunteers using Lymphoprep (Nycomed Pharma, Oslo, Norway) gradient centrifugation according to the manufacturer's instructions.

### Serial dilution of carcinoma cells

Serial dilution of KYSE 273 cells with normal PBMN was carried out to confirm the sensitivity of this RT-nested PCR assay. Known numbers of KYSE 273 cells were added to $1 \times 10^7$ normal PBMN, to give a ratio ranging from 1 : $10^2$ to 1 : $10^7$, then total RNA was extracted.

### RNA extraction

Total RNA was extracted from cell lines, PBMN, oesophageal tissues or lymph nodes using the acid guanidinium thiocyanate–phenol–chloroform extraction method (Chomczynski and Sacchi, 1987). Extracted RNA was dissolved in DEPC (diethylpyrocarbonate)-treated water and subjected to reverse transcription.

### Oligonucleotide primers

Primers were designed from the published sequence of human SCC antigen cDNA (Suminami, 1991). To avoid false-positive results caused by genomic DNA contamination, primers were selected which spanned introns (Figure 1). The outer primers produce a PCR fragment of 1083 bp. The nested primers produce a PCR fragment of 261 bp. The quality of RNA and cDNA synthesis was ascertained by amplification of glyceraldehyde phosphate dehydrogenase (GAPDH) gene as the internal control. The primer sequences for GAPDH primers were as follows: 5′-TGGTATCGTGGAAGGACTCATGAC-3′ and 5′-ATGCCAGT-GAGCTTCCCGTTCAGC-3′.

### RT-nested PCR

Reverse transcription was performed using a First Strand cDNA Synthesis kit (Pharmacia Biotech, Uppsala, Sweden) according to the manufacturer's instructions. The random hexamer was used as the RT primers. After RT, cDNA corresponding to 1 µg of RNA was used as the PCR template. The first-round PCR was performed in 50 µl reaction mix containing 20 mM Tris–HCl (pH 8.4), 50 mM potassium chloride (KCl), 2.0 mM magnesium chloride ($MgCl_2$), 1 µM each of the two primers (A and B), 0.2 mM dNTP and 1 U *Taq.* polymerase (Gibco-BRL, Grand Island, NY, USA). After initial heating at 94°C for 5 min, 35 cycles of PCR were carried out (94°C for 30 s, 50°C for 30 s and 72°C). For the nested PCR, 3 µl aliquot of the first-round PCR product was used as the template. The nested PCR was carried out in 50 µl reaction mix containing 20 mM Tris–HCl (pH 8.4), 50 mM KCl, 1.5 mM $MgCl_2$, 1 µM each of the two primers (C and D), 0.2 mM dNTP and 1 U *Taq.* polymerase. After initial heating at 94°C for 5 min, 30 cycles of PCR were carried out (94°C for 30 s, 60°C for 30 s and 72°C). For analysis, 10 µl of reaction product was run on a 2% agarose gel followed by SYBR green (Molecular Probe, Eugene, OR, USA) staining. The specificity of the PCR product was confirmed by restriction enzyme digestion pattern (*Eae* 1) and sequencing. The 261 bp RT-nested PCR products generated from KYSE273 cells were cloned into the pCR II plasmid vector using the TA cloning system (Invitrogen, Carlsbad, CA, USA) and sequenced by ALF autosequencer (Pharmacia Biotech). A Gen Bank-Update new sequences library nucleotide database search demonstrated that the sequence was specific to the SCCA1.

We took special care to avoid cross-contaminations. For example, we used filtertips of micropipettes in every procedure; preparation of PCR reagent and electrophoresis of PCR product were performed in separate places and a negative control, with no template cDNA, was run with every PCR to exclude carry-over contaminations.

*© 2000 Cancer Research Campaign*

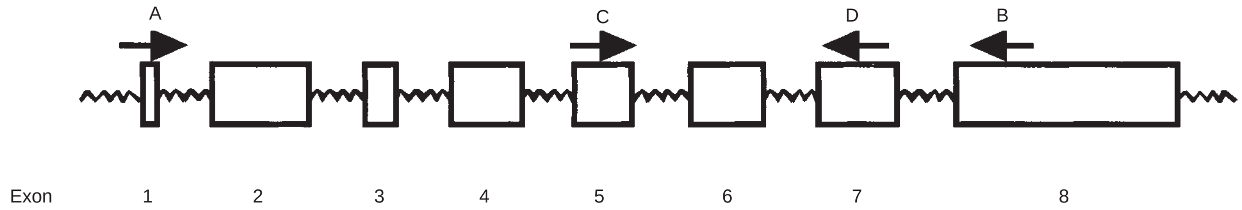

| | Position | Sequence | Product length |
|---|---|---|---|
| Outer primers | | | |
| A | 7 | 5'GCCCACCTCTGCTTCCTCTA3' | 1083 bp |
| B | 1070 | 5'GCTTCTGCTCCCTCCTCTGT3' | |
| Inner primers | | | |
| C | 470 | 5'GCAAATGCTCCAGAAGAAAG3' | 261 bp |
| D | 711 | 5'CGAGGCAAAATGAAAAGATG3' | |

**Figure 1** Schematic representation of the squamous cell carcinoma antigen gene and the position of primers used in reverse transcriptase-nested polymerase chain reaction. The first PCR was performed using primer A and B, followed by the nested PCR using primers C and D to obtain 1083 base-pair (bp) and 261-bp PCR product respectively. The positions of primers indicate their positions in the SCC antigen cDNA reported by Suminami et al (1991)

The results of RT-nested PCR were compared with that of pathological and immunohistochemical examination in a blind fashion.

### Immunohistochemistry

Paraffin-embedded sections were stained for epithelial cytokeratins by the streptavidin–peroxidase method. We used anti-cytokeratin antibody because the antibody for the SCC antigen is not commercially available. The antibody, AE1/AE3 (Dako, Kyoto, Japan), has been used for detection of occult tumour cells in lymph nodes (Greenson et al, 1994; Jeffers et al, 1994) and is known to be negative in normal lymph nodes. Sections were dewaxed, rehydrated and blocked endogenous peroxidase by 0.3% hydrogen peroxide in methanol for 30 min. Antigens were reactivated by microwave treatment. Cell membrane permeability was enhanced by 0.2% Triton X in phosphate-buffered saline (PBS) for 20 min. Non-specific antigen was blocked by 10% normal horse serum for 30 min at room temperature. Monoclonal murine antibody for epithelial cytokeratins, AE1/AE3 (Dako, Kyoto, Japan), in PBS containing 1% bovine serum albumin (BSA) was applied and sections were incubated 4°C overnight. Slides were washed 6 times in PBS and then incubated with biotinylated horse anti-mouse antibody for 40 min at room temperature. Biotinylated second antibody was detected by Vectastain Elite ABC Kit (Vector Laboratories, Burlingame, CA, USA) according to the manufacturer's instructions. The slides were then counterstained by haematoxylin. A lymph node with metastatic oesophageal SCC was included in all series as the positive control and a negative control run by exclusion of the primary antibody. All slides were evaluated in a blind fashion by two observers and all stained cells were evaluated by a pathologist. We excluded solitary stained cells without nuclear atypism and cell clusters that were located apparently out of the lymph node tissue.

### Statistical analysis

Wilcoxon signed-rank test was carried out to examine the difference among the histological examination, the immunohistochemical examination and the RT-nested PCR.

### RESULTS

### Sensitivity and specificity

Serial dilution experiments showed that our RT-nested PCR assay detected ten KYSE273 cells per $10^7$ of PBMN (Figure 2). We did not detect SCC antigen mRNA expression in 43 of the control lymph nodes from nine patients even when each PCR cycle was increased to 40 cycles (data not shown). Any non-specific band was not detected from RNA templates without RT (Figure 2).

### Clinical samples

We detected SCC antigen gene expression in all normal and malignant oesophageal tissues analysed in this study (data not shown). The result from all patients is shown in Table 2. Pathological examination, immunohistochemical examination and RT-nested PCR detected 36, 45 and 65 lymph nodes respectively. Twenty-six lymph nodes were positive only by RT-nested PCR. There were six metastases detected by immunohistochemistry but not by RT-nested PCR. In these six nodes, three were from a basaloid squamous carcinoma patient, two were from an adenocarcinoma patient and one node was from a SCC patient. RT-nested PCR detected significantly more lymph nodes than histological examination ($P < 0.0001$ by Wilcoxon signed-rank test) and immunohistochemical examination ($P = 0.0004$). The immunohistochemical examination either detected statistically more nodes than the pathological examination ($P = 0.0027$).

© 2000 Cancer Research Campaign

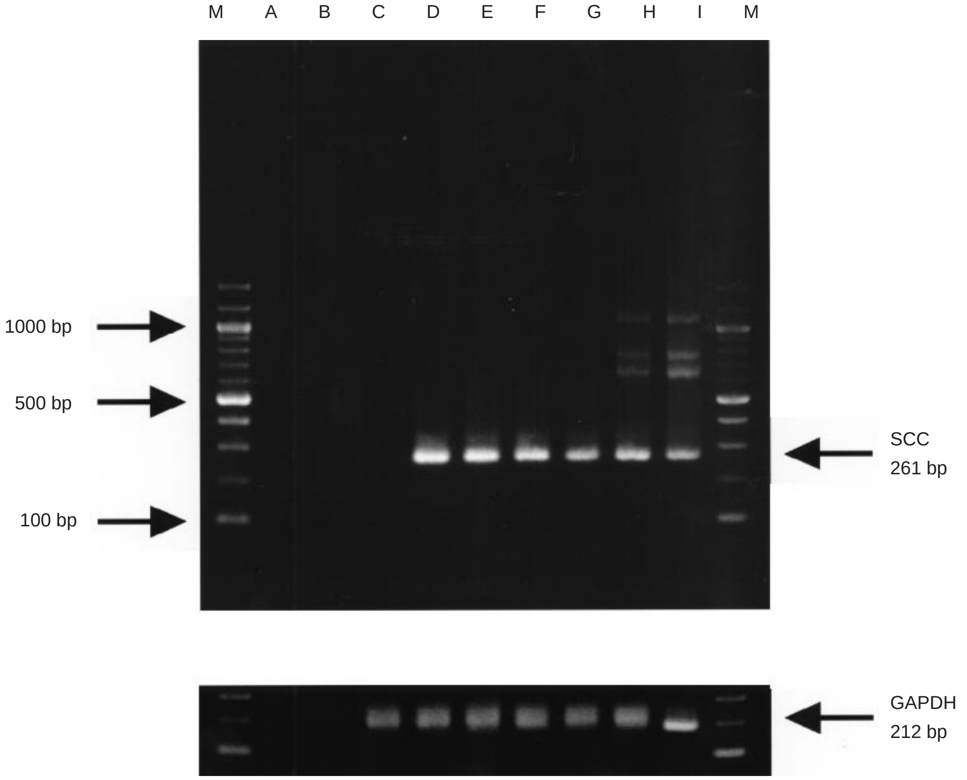

**Figure 2** Sensitivity of the assay. Serial dilution of KYSE273 cells in normal peripheral blood mononucleocytes (PBMN) was performed to confirm the sensitivity of our assay. Different numbers of KYSE273 cells were added to $10^7$ PBMN and then total RNA was extracted. The RT-nested PCR for SCC antigen gene constantly detected 10 of KYSE273 cells in $10^7$ of PBMN. Lane M: 100-bp ladder marker; lane A: the sample contained no cDNA; lane B: RNA from KYSE 273 cells without reverse transcription; lane C: 1 KYSE273 cells; lane D: 10 KYSE273 cells; lane E: $10^2$ KYSE273 cells; lane F: $10^3$ KYSE273 cells; lane G: $10^4$ KYSE273 cells; lane H: $10^5$ KYSE273 cells in $10^7$ of PBMN respectively, and lane I: KYSE273 cells. In lane H and I, 1083 bp of PCR product from outer primer set A and B, 724 bp of PCR product produced between primer A and D and 621 bp of PCR product produced between primer C and B are seen

**Table 2** Comparison of the detection of lymph nodes in all patients

| Pathology (+ = 36) | Immunohistochemistry (+ = 45) | RT-nested PCR (+ = 65) | Total (*n* = 620) |
|---|---|---|---|
| + | + | + | 33 |
| + | – | + | 0 |
| – | + | + | 6 |
| – | – | + | 26 |
| + | + | – | 3 |
| + | – | – | 0 |
| – | + | – | 3 |
| – | – | – | 549 |

**Table 3** Comparison of the detection of lymph nodes in SCC patients

| Pathology (+ = 25) | Immunohistochemistry (+ = 30) | RT-nested PCR (+ = 54) | Total (*n* = 536) |
|---|---|---|---|
| + | + | + | 25 |
| + | – | + | 0 |
| – | + | + | 4 |
| – | – | + | 25 |
| + | + | – | 0 |
| + | – | – | 0 |
| – | + | – | 1 |
| – | – | – | 481 |

In 12 squamous cell carcinoma cases, the RT-nested PCR detected all of pathologically positive nodes and all but one immunohistochemically positive node (Table 3). Both sensitivity and specificity of the RT-nested PCR were higher in SCC cases (98.2% and 99.8% respectively) than those in an adenocarcinoma case (84.6% and 90.9% respectively).

The influence of detected micrometastases on each patient is summarized in Table 4 (International Union Against Cancer, 1997). The RT-nested PCR for the SCC mRNA found micrometastases in 13 cases out of 14 cases whereas immunohistochemical examination detected five cases. In all pN0 patients, only RT-nested PCR revealed positive nodes near the primary tumour in each case. In three cases, nos 2, 3 and 14, RT-nested PCR detected tumour dissemination beyond regional lymph nodes (Figure 3) (Japanese Society for Esophageal Diseases, 1992). Especially in case 14, who was pN0 by pathological and immunohistochemical examinations, RT-nested PCR indicated the spread of cancer cells toward the para-aortic lymph node via the perigastric lymph node.

In case 10, a poorly differentiated adenocarcinoma, RT-nested PCR detected eight nodes out of nine pathologically positive nodes and two more positive nodes that were not detected pathologically. In contrast to case 10, in case 7, a basaloid squamous, RT-nested PCR for SCC antigen gene did not detect any positive nodes out of 51 dissected nodes.

 © 2000 Cancer Research Campaign

**Table 4** Influence of the detection of micrometastases

| | Number of lymph nodes Pathology/IH/RT-nested PCR/Total | | | | | | Influence on the N, M and stage Pathology/IH/RT | | |
|---|---|---|---|---|---|---|---|---|---|
| **No.** | **Cervical** | **Mediastinal** | **Perigastric** | **Coeliac and para-aortic** | **Total** | **pT** | **N** | **M** | **Stage** |
| 1 | 3/3/4/20 | 0/0/0/0 | 0/0/0/11 | 0/0/0/0 | 3/3/4/31 | pT2 | 1/1/1 | 0/0/0 | 2B/2B/2B |
| 2 | 2/2/2/34 | 0/0/1/13 | 0/0/0/2 | 0/0/0/0 | 2/2/3/49 | pT4 | 1/1/1 | 0/0/1 | 3/3/4 |
| 3 | 4/5/10/31 | 0/0/2/8 | 0/0/0/0 | 0/0/0/0 | 4/5/12/39 | pT2 | 1/1/1 | 0/0/1 | 2B/2B/4 |
| 4 | 3/6/6/27 | 3/3/4/26 | 1/1/2/18 | 0/0/0/0 | 7/10/12/71 | pT3 | 1/1/1 | 1a/1a/1a | 4A/4A/4A |
| 5 | 0/0/0/0 | 0/0/1/7 | 0/0/0/19 | 0/0/0/0 | 0/0/1/26 | pT1 | 0/0/1 | 0/0/0 | 1/1/2B |
| 6 | 0/0/0/0 | 0/0/0/10 | 1/2/2/19 | 0/0/0/4 | 1/2/2/33 | pT1 | 1/1/1 | 0/0/0 | 2B/2B/2B |
| 7 | 0/0/0/15 | 2/2/0/14 | 0/1/0/22 | 0/0/0/0 | 2/3/0/51 | pT3 | 1/1/0 | 0/0/0 | 3/3/2A |
| 8 | 0/0/0/0 | 1/1/1/1 | 4/4/5/14 | 0/0/0/0 | 5/5/6/15 | pT3 | 1/1/1 | 0/0/0 | 3/3/3 |
| 9 | 0/0/0/0 | 0/0/0/11 | 0/0/1/28 | 0/0/0/0 | 0/0/1/39 | pT3 | 0/0/1 | 0/0/0 | 2A/2A/3 |
| 10 | 0/0/0/0 | 4/5/6/11 | 5/7/5/18 | 0/0/0/4 | 9/12/11/33 | pT3 | 1/1/1 | 0/0/0 | 3/3/3 |
| 11 | 0/0/0/0 | 1/1/1/27 | 0/0/3/32 | 0/0/0/7 | 1/1/4/66 | pT2 | 1/1/1 | 0/0/0 | 2B/2B/2B |
| 12 | 0/0/0/0 | 0/0/0/18 | 1/1/2/20 | 0/0/0/0 | 1/1/2/38 | pT2 | 1/1/1 | 0/0/0 | 2B/2B/2B |
| 13 | 0/0/0/0 | 0/0/0/29 | 1/1/2/38 | 0/0/0/0 | 1/1/2/67 | pT2 | 1/1/1 | 0/0/0 | 2B/2B/2B |
| 14 | 0/0/0/0 | 0/0/1/25 | 0/0/3/31 | 0/0/1/6 | 0/0/5/62 | pT2 | 0/0/1 | 0/0/1a | 2A/2A/4A |

IH: immunohistochemical examination, RT: RT-nested PCR.

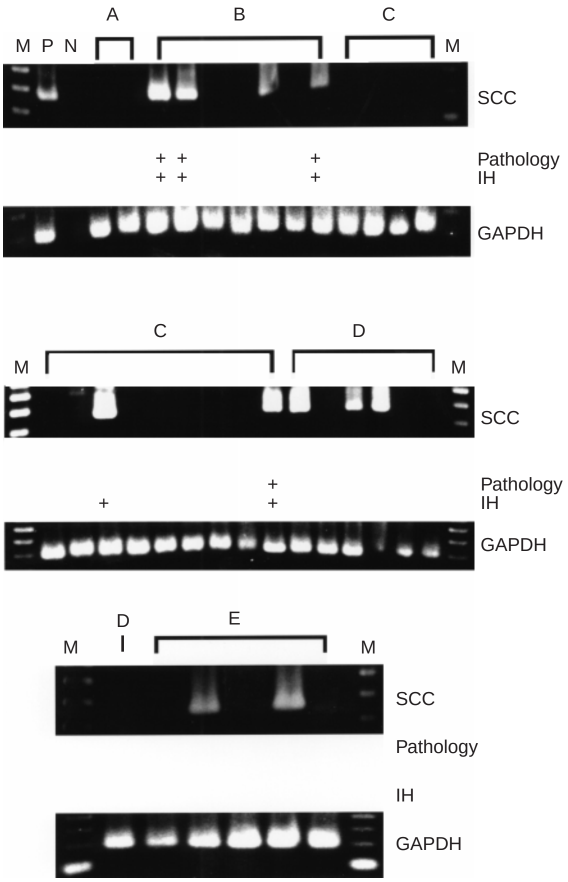

**Figure 3** Representative electrophoresis pattern of case 3. In this case, pathological examination, immunohistochemical examination (IH) and RT-nested PCR detected 4, 5 and 12 lymph nodes respectively. Only RT-nested PCR revealed mediastinal lymph node metastases. The first, ninth and tenth lanes of C and the third lane of E include two lymph nodes. The first lane of E includes three lymph nodes. M: 100 bp ladder marker, P: KYSE273, N: negative control contained no template cDNA, A: superficial cervical lymph nodes, B: cervical paraoesophageal lymph nodes, C: deep cervical lymph nodes, D: supraclavicular lymph nodes, E: thoracic paraoesophageal lymph nodes. These names of lymph nodes are according to the nomenclature of the Japanese Society for Esophageal Disease (JSED)

The pN classification by the RT-nested PCR advanced in three cases and dropped in a basaloid SCC case. The pM classification advanced in three cases by the RT-nested PCR. Finally, the pTNM stage advanced in five cases and dropped in a basaloid SCC case by the RT-nested PCR.

The average micrometastases were 1.9 nodes a case. There were four cases that had more micrometastases than the average. We are prospectively following the impact of micrometastases on the prognosis.

### DISCUSSION

The lymph node metastasis, as well as the depth of penetration (Skinner et al, 1982), and the surgical curability (Roder et al, 1994), is regarded as an important prognostic factor in oesophageal cancer (Sugimachi et al, 1986; Siewert and Roder, 1992; Kato et al, 1993; Baba et al, 1994, 1997; Fahn et al, 1994; Nishimaki et al, 1994; Bhansali et al, 1997). Recent development of molecular biology makes it possible to detect occult tumour cells and the influence of occult tumour cells has become evident. Izbicki et al (1997) revealed the impact of micrometastases for the prognosis of oesophageal cancer patients by immunohistochemistry using epithelial-specific antibody, Ber-EP4. They reported that Ber-EP4-positive cells in ‘tumour free’ lymph nodes were independently predictive of significantly reduced relapse-free survival and overall survival. Luketich et al (1998) used RT-PCR for carcinoembryonic antigen (CEA) mRNA to detect occult lymph node metastasis of oesophageal cancer and reported that a positive RT-PCR with negative histological findings may have poor prognostic implications. On the other hand, McGuckin et al (1996) analysed 208 axillary node-negative breast cancer patients using a combination of limited step-sectioning and immunohistochemical staining (with cytokeratin (MNF.116) and MUC1 (BC2) antibodies) and, though they found that the presence and increasing size of occult nodal metastases were significantly associated with poorer disease-free survival, occult nodal metastases did not affect overall survival. Glickman et al (1999) reported that they did not find any correlation between poorer survival rates and

© 2000 Cancer Research Campaign occult lymph node metastases in 78 of lymph node-negative oesophageal patients using serial sectioning and immunohistochemistry (anti-cytokeratin antibody; AE1/AE3). Thus it seems likely that occult lymph node metastasis affects disease-free survival, but it is still controversial whether it really has an impact on overall survival. Larger scale and longer period study is desirable to clarify this issue.

The SCC antigen was isolated from a metastatic, cervical SCC (Kato and Torigoe, 1977) and has been widely applied as a serum tumour marker of SCC (Kato, 1992). On the other hand, it also exists in normal squamous epithelium (Maruo et al, 1985; Matsuda et al, 1990). The SCC antigen gene was cloned in 1991 and was identified to be a member of ovalbumin family of serine protease inhibitors (Suminami, 1991) and it was revealed to be located on the locus at 18q21.3 and to contain a tandem array of two genes, SCCA1 and SCCA2 (Schneider et al, 1995). These two genes are thought to code for the neutral and acidic forms of the protein respectively. It is thought that the neutral form of the SCC antigen exists in both normal and malignant squamous epithelium. On the other hand, the acidic form is the dominant form of the serum SCC and it is thought to be secreted by malignant cells. However, the sensitivity of the serum SCC antigen is 28.6–37% (Mealy et al, 1996; Nakamura et al, 1998; Quillien et al, 1998) in oesophageal cancer. Therefore the acidic form may not always be produced in oesophageal cancer cells. We think the SCCA1 gene is suitable for the RT-PCR target because it already exists in squamous cells.

Our RT-nested PCR for the SCC antigen gene more efficiently detected micrometastases of oesophageal cancer than the immunohistochemical staining for the cytokeratins (AE1/AE3). It is theoretically possible that the RT-nested PCR overlooks micrometastases when small numbers of cancer cells are included exclusively in the other half of the lymph node. The only lymph node from a SCC case that was negative by RT-nested PCR but positive by immunohistochemical examination (Table 3) may be the case. Though this problem may occur equally with each method, RT-nested PCR found 25 micrometastases that were not detected by immunohistochemical examination. Generally, in such cases where a small number of micrometastatic cells are found, RT-nested PCR which can analyse a lymph node en bloc which has an advantage.

Clinical application of these sensitive but laborious methods is still a problem. Indeed it is not practical to examine every dissected lymph node by RT-PCR. In order to use these methods practically, it may be better to focus some key lymph nodes, for example, recurrent nerve chain (Malassagne et al, 1997) or perigastric nodes or, as Stenman et al (1997) indicated, these methods can be applicable to detect circulating tumour cells. These highly sensitive methods can provide new clues to select patients at risk or to investigate the way of tumour spread.

We detected SCC antigen gene expression in an adenocarcinoma case. This means, although sensitivity in adenocarcinoma seems lower than that in SCC, even adenocarcinomas may express SCC antigen mRNA in the oesophagus. Though the expression of the SCC antigen mRNA in adenocarcinomas has not been fully investigated, some previous reports support our findings. For example, Ueda et al (1984) reported that the SCC antigen (referred to as TA-4) was detected in some adenocarcinomas of the uterine cervix and less frequently in those of the endometrium. Crombach et al (1989) showed elevated cytosolic SCC antigen protein in adenocarcinomas of the uterine cervix in comparison with other gynaecological adenocarcinomas. Takeshima et al (1992) detected mRNA of SCC antigen in the columnar cells of the cervix but not in the endometrium. Considering these findings, combined with our observations, we expect that we can detect occult metastases of an oesophageal adenocarcinoma using SCC antigen gene expression. However, because we have experienced only two cases other than SCCs, further study will be needed for the application of SCC antigen gene expression for detecting oesophageal adenocarcinoma micrometastasis.

On the other hand, in a basaloid squamous carcinoma case, our RT-nested PCR did not detect any metastases. The primary tumour of this case had a small amount of SCC; most of the tumour was basaloid carcinoma cells. The lymph node metastases of this case exclusively consisted of these basaloid carcinoma cells. This may be the reason our RT-nested PCR detected SCC antigen expression in the primary tumour but not in lymph node metastases.

In conclusion, the SCC antigen gene is a useful target to find occult oesophageal SCC cells. The RT-nested PCR for the SCC antigen gene seems more sensitive in SCC cases. This unique characteristic of the SCC antigen gene differs from CEA or cytokeratins. As Stenman et al (1997) suggested in cervical cancer cases, this method will be applicable in other SCCs.

### ACKNOWLEDGEMENTS

This work was supported in part by grants 09671301(C) and 09470265(B) from the Ministry of Education, Culture, and Science of Japan. We would like to thank Prof. H Yamabe (Laboratory of Anatomic Pathology, Kyoto University Hospital) for valuable advice and teaching on histological findings.

### REFERENCES

Abe S, Tachibana M, Shiraishi M and Nakamura T (1990) Lymph node metastasis in resectable esophageal cancer [see comments]. *J Thorac Cardiovasc Surg* **100**: 287–291

Baba M, Aikou T, Yoshinaka H, Natsugoe S, Fukumoto T, Shimazu H and Akazawa K (1994) Long-term results of subtotal esophagectomy with three-field lymphadenectomy for carcinoma of the thoracic esophagus [see comments]. *Ann Surg* **219**: 310–316

Baba M, Aikou T, Natsugoe S, Kusano C, Shimada M, Kimura, S and Fukumoto T (1997) Lymph node and perinodal tissue tumor involvement in patients with esophagectomy and three-field lymphadenectomy for carcinoma of the esophagus. *J Surg Oncol* **64**: 12–16

Bhansali MS, Fujita H, Kakegawa T, Yamana H, Ono T, Hikita S, Toh Y, Fujii T, Tou U and Shirouzu K (1997) Pattern of recurrence after extended radical esophagectomy with three-field lymph node dissection for squamous cell carcinoma in the thoracic esophagus. *World J Surg* **21**: 275–281

Chomczynski P and Sacchi N (1987) Single-step method of RNA isolation by acid guanidinium thiocyanate-phenol-chloroform extraction. *Anal Biochem* **162**: 156–159

Crombach G, Scharl A, Vierbuchen M, Wurz H and Bolte A (1989) Detection of squamous cell carcinoma antigen in normal squamous epithelia and in squamous cell carcinomas of the uterine cervix. *Cancer* **63**: 1337–1342

Fahn HJ, Wang LS, Huang BS, Huang MH and Chien KY (1994) Tumor recurrence in long-term survivors after treatment of carcinoma of the esophagus. *Ann Thorac Surg* **57**: 677–681

Glickman JN, Torres C, Wang HH, Turner JR, Shahsafaei A, Richards WG, Sugarbaker DJ and Odze RD (1999) The prognostic significance of lymph node micrometastasis in patients with esophageal carcinoma. *Cancer* **85**: 769–778

Greenson JK, Isenhart CE, Rice R, Mojzisik C, Houchens D and Martin EW, Jr (1994) Identification of occult micrometastases in pericolic lymph nodes of Duke's B colorectal cancer patients using monoclonal antibodies against cytokeratin and CC49. Correlation with long-term survival. *Cancer* **73**: 563–569

International Union Against Cancer (1997) *TNM Classification of Malignant Tumours*. Wiley-Liss: New York

© 2000 Cancer Research Campaign

Izbicki JR, Hosch SB, Pichlmeier U, Rehders A, Busch C, Niendorf A, Passlick B, Broelsch CE and Pantel K (1997) Prognostic value of immunohistochemically identifiable tumor cells in lymph nodes of patients with completely resected esophageal cancer. *N Engl J Med* **337**: 1188–1194

Japanese Society for Esophageal Diseases (1992) *Guidelines for the Clinical and Pathologic Studies on Carcinoma of the Esophagus*. Kanehara: Tokyo

Jeffers MD, O'Dowd GM, Mulcahy H, Stagg M, O'Donoghue DP and Toner M (1994) The prognostic significance of immunohistochemically detected lymph node micrometastases in colorectal carcinoma. *J Pathol* **172**: 183–187

Kato H (1992) Squamous cell carcinoma antigen. In: *Serological Cancer Markers,* Sell S (ed), pp. 437–451. Totowa: Humana

Kato H and Torigoe T (1977) Radioimmunoassay for tumor antigen of human cervical squamous cell carcinoma. *Cancer* **40**: 1621–1628

Kato H, Tachimori Y, Mizobuchi S, Igaki H and Ochiai A (1993) Cervical, mediastinal, and abdominal lymph node dissection (three-field dissection) for superficial carcinoma of the thoracic esophagus. *Cancer* **72**: 2879–2882

Law SY, Fok M and Wong J (1996) Pattern of recurrence after oesophageal resection for cancer: clinical implications. *Br J Surg* **83**: 107–111

Luketich JD, Kassis ES, Shriver SP, Nguyen NT, Schauer PR, Weigel TL, Yousem SA and Siegfried JM (1998) Detection of micrometastases in histologically negative lymph nodes in esophageal cancer. *Ann Thorac Surg* **66**: 1715–1718

McGuckin MA, Cummings MC, Walsh MD, Hohn BG, Bennett IC and Wright RG (1996) Occult axillary node metastases in breast cancer: their detection and prognostic significance. *Br J Cancer* **73**: 88–95

Malassagne B, Tiret E, Duprez D, Coste J, de Sigalony JP and Parc R (1997) Prognostic value of thoracic recurrent nerve nodal involvement in esophageal squamous cell carcinoma. *J Am Coll Surg* **185**: 244–249

Maruo T, Shibata K, Kimura A, Hoshina M and Mochizuki M (1985) Tumor-associated antigen, TA-4, in the monitoring of the effects of therapy for squamous cell carcinoma of the uterine cervix. Serial determinations and tissue localization. *Cancer* **56**: 302–308

Matsuda H, Mori M, Tsujitani S, Ohno S, Kuwano H and Sugimachi K (1990) Immunohistochemical evaluation of squamous cell carcinoma antigen and S-100 protein-positive cells in human malignant esophageal tissues. *Cancer* **65**: 2261–2265

Mealy K, Feely J, Reid I, McSweeney J, Walsh T and Hennessy TP (1996) Tumour marker detection in oesophageal carcinoma. *Eur J Surg Oncol* **22**: 505–507

Morita M, Kuwano H, Ohno S, Furusawa M and Sugimachi K (1994) Characteristics and sequence of the recurrent patterns after curative esophagectomy for squamous cell carcinoma. *Surgery* **116**: 1–7

Nakamura T, Ide H, Eguchi R, Hayashi K, Takasaki K and Watanabe S (1998) CYFRA 21–1 as a tumor marker for squamous cell carcinoma of the esophagus. *Dis Esophagus* **11**: 35–39

Nishimaki T, Tanaka O, Suzuki T, Aizawa K, Hatakeyama K and Muto T (1994) Patterns of lymphatic spread in thoracic esophageal cancer. *Cancer* **74**: 4–11

Pelkey TJ, Frierson HF Jr and Bruns DE (1996) Molecular and immunological detection of circulating tumor cells and micrometastases from solid tumors. *Clin Chem* **42**: 1369–1381

Quillien V, Raoul JL, Laurent JF, Meunier B and Le Prise E (1998) Comparison of Cyfra 21–1, TPA and SCC tumor markers in esophageal squamous cell carcinoma. *Oncol Rep* **5**: 1561–1565

Raj GV, Moreno JG and Gomella LG (1998) Utilization of polymerase chain reaction technology in the detection of solid tumors. *Cancer* **82**: 1419–1442

Roder JD, Busch R, Stein HJ, Fink U and Siewert JR (1994) Ratio of invaded to removed lymph nodes as a predictor of survival in squamous cell carcinoma of the oesophagus. *Br J Surg* **81**: 410–413

Schneider SS, Schick C, Fish KE, Miller E, Pena JC, Treter SD, Hui SM and Silverman GA (1995) A serine proteinase inhibitor locus at 18q21.3 contains a tandem duplication of the human squamous cell carcinoma antigen gene. *Proc Natl Acad Sci USA* **92**: 3147–3151

Shimada Y (1999) Esophageal cancers. In: *Human Cell Culture,* Masters JRW and Palsson B (eds), Vol. 1, pp. 179–212. Kluwer Academic: Amsterdam

Shimada Y, Imamura M, Wagata T, Yamaguchi N and Tobe T (1992) Characterization of 21 newly established esophageal cancer cell lines [published erratum appears in *Cancer* 1992 **70**(1): 206]. *Cancer* **69**: 277–284

Siewert JR and Roder JD (1992) Lymphadenectomy in esophageal cancer surgery. *Dis Esophagus* **5**: 91–97

Skinner DB, Dowlatshahi KD and DeMeester TR (1982) Potentially curable cancer of the esophagus. *Cancer* **50**: 2571–2575

Stenman J, Lintula S, Hotakainen K, Vartiainen J, Lehvaslaiho H and Stenman UH (1997) Detection of squamous-cell carcinoma antigen-expressing tumour cells in blood by reverse transcriptase-polymerase chain reaction in cancer of the uterine cervix. *Int J Cancer* **74**: 75–80

Sugimachi K, Matsuura H, Kai H, Kanematsu T, Inokuchi K and Jingu K (1986) Prognostic factors of esophageal carcinoma: univariate and multivariate analyses. *J Surg Oncol* **31**: 108–112

Suminami Y, KF, Sekiguchi K and Kato H (1991) Squamous cell carcinoma antigen is a new member of the serine protease inhibitors. *Biochem Biophys Res Commun* **181**: 51–58

Takeshima N, Suminami Y, Takeda O, Abe H, Okuno N and Kato H (1992) Expression of mRNA of SCC antigen in squamous cells. *Tumour Biol* **13**: 338–342

Ueda G, Inoue Y, Yamasaki M, Inoue M, Tanaka Y, Hiramatsu K, Saito J, Nishino T and Abe Y (1984) Immunohistochemical demonstration of tumor antigen TA-4 in gynecologic tumors. *Int J Gynecol Pathol* **3**: 291–298

*© 2000 Cancer Research Campaign*
